# The Performance of Immunoassays to Measure Antibodies to the *Chlamydia trachomatis* Antigen Pgp3 in Different Epidemiological Settings for Trachoma

**DOI:** 10.4269/ajtmh.21-0541

**Published:** 2021-08-16

**Authors:** Sarah Gwyn, Andrew W. Nute, Eshetu Sata, Zerihun Tadesse, Ambahun Chernet, Mahteme Haile, Taye Zeru, Danaya Bethea, Christian Laurent, E. Kelly Callahan, Scott D. Nash, Diana L. Martin

**Affiliations:** ^1^ Division of Parasitic Diseases and Malaria, Centers for Disease Control and Prevention, Atlanta, Georgia;; ^2^ The Carter Center, Atlanta, Georgia;; ^3^ The Carter Center, Ethiopia;; ^4^ Amhara Public Health Institute, Ethiopia

## Abstract

Programs to eliminate trachoma as a public health problem use prevalence of the clinical sign trachomatous inflammation—follicular (TF) in 1- to 9-year-olds in endemic districts to make decisions to begin or end mass drug administration with azithromycin. Trachomatous inflammation—follicular is used as a proxy for transmission of ocular *Chlamydia trachomatis* infection. Long-term monitoring of previously endemic districts for recrudescence of ocular *C. trachomatis* infection would benefit from a simple blood test that could be integrated with other public health programs. In this study, we evaluated multiple tests to measure antibodies against the *C. trachomatis* antigen Pgp3—a multiplex bead assay (MBA), an ELISA, and two versions of a lateral flow assay (LFA)—in four districts of the Amhara region of Ethiopia with varying levels of TF. Seroprevalence and seroconversion rate (SCR) results were proportional to TF prevalence by district for most tests, with the notable exception of the LFA using colloidal gold as the developing reagent. Changing the test developing reagent to black latex improved agreement between serological measures and TF prevalence and in inter-rater agreement. Seroconversion rate estimates using data derived from the LFA-gold assay were inconsistent with the shape of the age-seroprevalence curve, which did not increase in older ages. These data revealed potential complications with using SCR that will need further evaluation. Data from MBA, ELISA, and LFA with the black test line showed good agreement with each other and proportionality to TF estimates, providing further data that serology has potential utility for trachoma surveillance.

## INTRODUCTION

Trachoma, the leading infectious cause of blindness, is an eye disease caused by repeated ocular infection with the bacterium *Chlamydia trachomatis* (Ct) and is targeted for elimination as a public health problem (EPHP).^1^ The EPHP is achieved through several interventions, called the SAFE strategy, including surgery to correct trichiasis, mass drug administration of antibiotics, facial cleanliness, and environmental improvements. The EPHP target of < 5% of children aged 1 to 9 years with trachomatous inflammation—follicular (TF) allows for low levels of transmission of Ct to occur even after EPHP has been validated. Tools to monitor for recrudescence of infection will be essential upon cessation of interventions once countries achieve EPHP targets.^2^

Antibodies against Ct antigens show potential as a surveillance tool in postelimination settings.^2^^–^^5^ Although not diagnostic of infection in an individual, the presence of antibodies indicates exposure to Ct and can show transmission trends in a community.^6^ It is still unclear how serological results should be used to detect potential recrudescence of infection. Preliminary models suggest that a mean seroprevalence of less than 6.2% and a seroconversion rate (SCR) of below 0.015 seroconversion events per year in 1- to 9-year-olds correspond to a TF of less than 5%.^7^ More data are needed from a variety of epidemiological settings to understand the relationship between TF, SCR, and seroprevalence, and to define appropriate seropositivity thresholds for programs to maintain EPHP.^2^

There are three platforms available to measure antibodies against the immunodominant Ct antigen Pgp3—multiplex bead assay (MBA), ELISA, and lateral flow assay (LFA)—that were developed to be responsive to the needs of national programs.^8^^–^^10^ As the evidence base to understand the utility of serology for population-based trachoma surveillance is being developed through operational research, the antibody tests have been refined and improved. The ELISA has been recently modified to a double antigen format to improve assay performance, similar to other Pgp3 ELISAs.^11^ The LFA has undergone several iterations, starting out in a cassette for use in house-to-house surveys,^10^^,^^12^ moving to laboratory-based “dipstick” format,^13^ the most recent version using a black latex detector to improve readability.^14^ Each time a new version is developed, testing must be performed to ensure results are consistent between platforms.

In 2017, a survey was conducted in four districts in the Amhara region of Ethiopia with historically different trachoma endemicity^15^; during which dried blood spots (DBS) were collected to characterize antibody responses within populations experiencing different levels of trachoma transmission. Within that survey, we nested a substudy to compare the performance of the different platforms to measure antibodies against Pgp3. Here, we compare seroprevalence and SCR estimates between the MBA, two versions of the LFA (one using a colloidal gold detecting reagent and one using a black latex detection reagent) and a newly developed ELISA using a double antigen format in the four districts. We also evaluate the performance of the newly developed LFA and ELISA by assessing inter-rater agreement for the LFA and agreement between each new assay and the established MBA.

## METHODS

### Ethics.

The study protocol was approved by the Emory University IRB (protocol 079-2006), the Amhara Regional Health Bureau and the Federal Ministry of Science and Technology of Ethiopia. Staff from the CDC did not have contact with study participants or access to identifying information and were determined to be not engaged in human subjects research.

### Study sites.

Population-based prevalence impact and surveillance surveys were conducted in four districts (Alefa, Andabet, Dera, and Woreta Town) in the Amhara region of Ethiopia as previously described.^15^ Briefly, a multistage cluster-random survey was conducted in all four districts. All individuals aged 1 year and older in the selected households were invited to participate in the survey. Dried blood spots from only 1 to 9-year-olds (*N* = 2195) were tested by each assay described below. Of these individuals, 1,055 (48.1%) were male. The prevalence of TF for each district is illustrated in Table 2. The prevalence of ocular Ct infection among 1 to 5-year-olds was 0 in all districts expect for Andabet, where it was 11.3%.

### Dried blood spot collection.

Retractable lancets were used to collect finger prick blood onto filter paper (TropBio Pty Ltd, Townsville, Queensland, Australia) containing six blood spots extensions, each holding approximately 10 µL of blood. Each filter paper was labeled with a barcode, scanned into the survey software, air-dried for at least 2 hours, and then placed into a sealable plastic bag. Filter papers were stored in coolers in the field, then stored at −20°C at the Amhara Public Health Institute until they were shipped to CDC in the United States at ambient temperature.^15^ All serologic testing was performed at CDC.

### Multiplex bead assay.

Sera eluted from DBS were tested by MBA for antibodies to the antigen Pgp3 as reported in the parent study by Nash et al.^15^ The cutoff median fluorescence intensity minus background (MFI-bg) for positivity was 1,558.

### Double antigen ELISA.

Immulon 2HB plates (Thermo Fisher Scientific, Waltham, MA) were coated with 50 µL of 300 ng/mL of Pgp3 antigen in NaCO_3_ pH 9.6 overnight at 4°C. The next day, wells were washed four times with PBST (0.3% Tween 20 in phosphate-buffered saline [PBS]) and incubated for 30 minutes with 100 µL of StabilCoat^®^ (SurModics, Eden Prairie, MN). Buffer was decanted from wells and plates were dried in a vacuum oven at 30–40°C for 4 hours. Dried plates were stored in foil packages with a 1 g sachet of desiccant at 4°C until use. Each dried blood spot extension was eluted overnight at 4°C in 250 µL PBST containing 5% milk powder (PBST-milk) for a final serum dilution of 1:50. Diluted sample (50 µL) was added to each well of the Pgp3-coated ELISA plate and incubated for 2 hours. Wells were washed four times with 200 µL of PBST. Pgp3-HRP (Expedeon, San Diego, CA) was diluted 1:5,000 in PBST and 50 µL was added to each well to detect any bound anti-Pgp3 immunoglobulin. After a 1 hour incubation, plates were washed four times in 200 µL of PBST. 3,3′,5,5′-Tetramethylbenzidine (TMB) was added (50 µL/well) and incubated for 10 minutes. To stop the reaction, 50 µL 1M H_2_SO_4_ was added per well. The plates were read immediately at 450 nm on a microplate reader (BioTek, Winooski, VT). A set of standards (1,000U, 500U, 200U, and 50U) along with negative human serum (NHS) and a blank (PBST-milk) were run on each plate. The absorbance value of the blank well was subtracted from the absorbance value of each sample well (A-blank). The A-blank value of each sample was normalized to the 500U standard (Norm A-blank). The cutoff of positivity was a Norm A-blank of 0.095 as determined by receiver operating curve (ROC) analysis with a panel of 74 nonendemic negatives and 77 samples previously testing positive by MBA.

### Lateral flow dipstick assays.

Dried blood spots were tested by a lateral flow dipstick assay with a colloidal gold detecting reagent conjugated to Pgp3 (LFA-gold; Abcam, Cambridge, United Kingdom) and a black latex detecting reagent conjugated to Pgp3 (LFA-latex; Abcam) as described.^10^^,^^14^ All samples tested on LFA-latex and samples from Alefa tested on LFA-gold were read by two readers to assess inter-rater agreement.

### Statistical analysis.

Seroconversion rates were calculated using a generalized linear model with a complementary log-log link and robust standard errors. The model assumed constant force of infection and no seroreversion. The analyses were performed in R statistical program (https://www.r-project.org/) as described^15^ and were confirmed on multiple runs. Inter-rater agreement for LFA testing and agreement between tests were quantified by kappa in GraphPad Prism (Graphpad Software, San Diego, CA).

## RESULTS

### Antibody testing and prevalence.

Of 2,195 DBS tested overall, all were tested by MBA, 2,186 were tested by LFA (gold and latex), and 2,135 were tested by ELISA across the four survey districts (Table 1). The districts that had a TF prevalence > 5% (Andabet and Dera) at the time of the survey had a seroprevalence > 10% for all assays (Table 2). The districts that had a TF prevalence < 5% (Alefa and Woreta Town) had a seroprevalence < 10% for all assays (Table 2). For Alefa, all assays except for LFA-gold had a seroprevalence < 5%. TF data and antibody prevalence by each test is illustrated in Table 2.

**Table 1:** The number of tests run on each assay by district

EU	MBA	LFA (gold and latex)	ELISA
Alefa	712	703	660
Andabet	580	580	579
Woreta Town	275	275	270
Dera	628	628	626
Total	2,195	2,186	2,135

EU = evaluation unit; LFA = lateral flow assay; MBA = multiplex bead assay.

**Table 2:** TF and age-adjusted seroprevalence for each assay by district. 95% CI in parenthesis

EU	TF (95% CI)	MBA (95% CI)	LFA-gold (95% CI)	LFA-latex (95% CI)	ELISA (95% CI)
Alefa	3.2% (1.4–5.7)	1.1% (0.4–6.6)	9.6% (4.8–18.4)	2.5% (0.8–8.9)	1.8% (0.6–8.1)
Andabet	37.0% (31.1–43.3)	37.1% (27.0–48.7)	37.6% (27.3–49.4)	41.6% (31.2–53)	39.8% (29.6–51.2)
Woreta Town	2.7% (1.5–4.5)	5.0% (1.4–19.5)	8.0% (3.0–23.2)	6.3% (2.0–21.1)	6.5% (1.8–21.8)
Dera	14.7% (10.0–20.5)	11.6% (6.1–21.2)	19.0% (11.6–29.8)	13.5% (7.6–23.4)	12.4% (6.7–22.1)

EU = evaluation unit; LFA = lateral flow assay; MBA = multiplex bead assay; TF = trachomatous inflammation—follicular.

**Figure 1.**
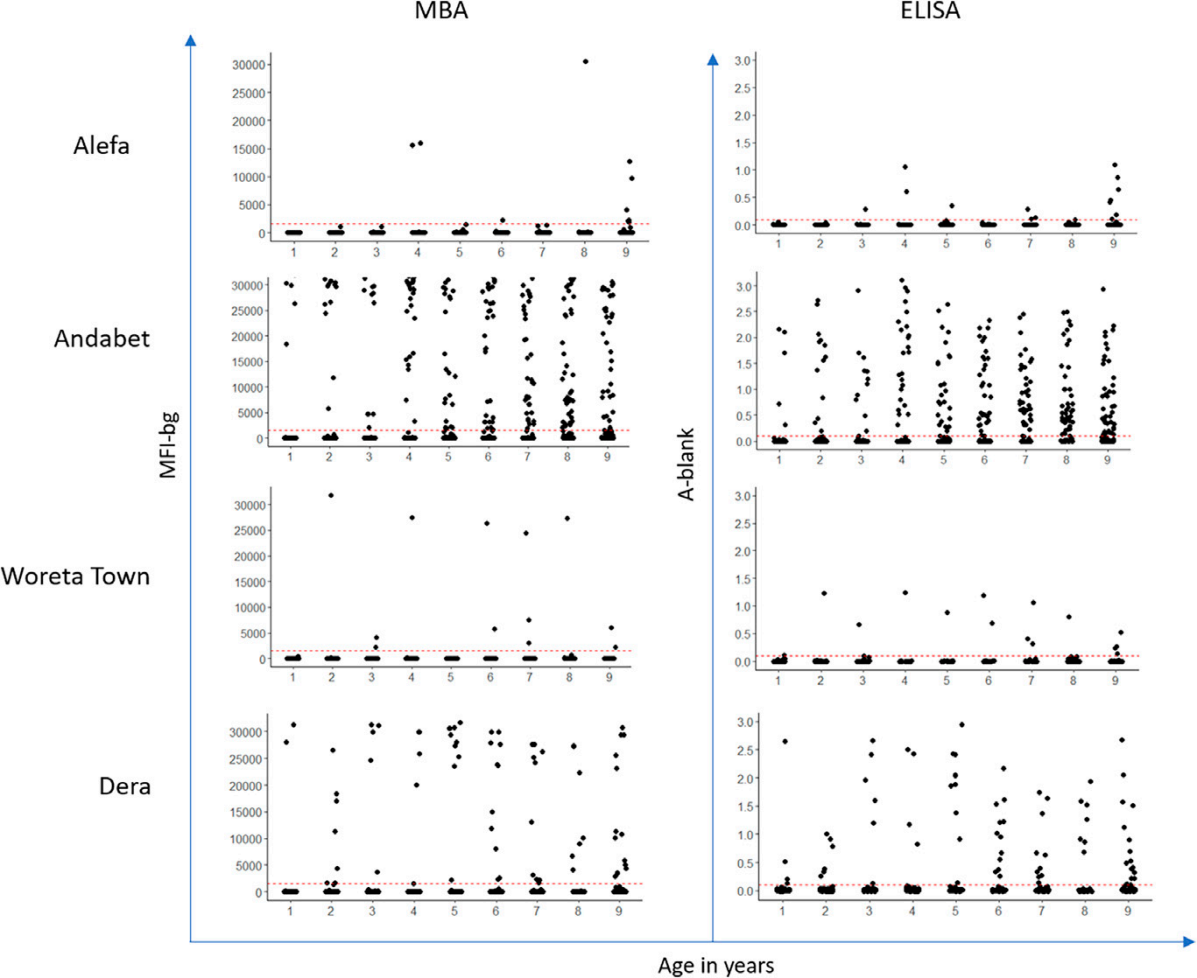
Intensity of antibody response, represented by median fluorescence intensity with background subtracted (MFI-bg) by year of age for each evaluation unit. A-blank = absorbance − blank; LFA = lateral flow assay; MBA = multiplex bead assay. This figure appears in color at www.ajtmh.org.

Few specimens (five or fewer) from districts with TF prevalence < 5% (Alefa and Woreta Town) had high-intensity responses by MBA or ELISA, whereas the majority of the antibody-positive specimens from districts with TF prevalence > 5% (Andabet and Dera) had MFI-bg or absorbances at the upper range of the assay (Figure 1).

### Seroconversion rate estimates.

**Figure 2.**
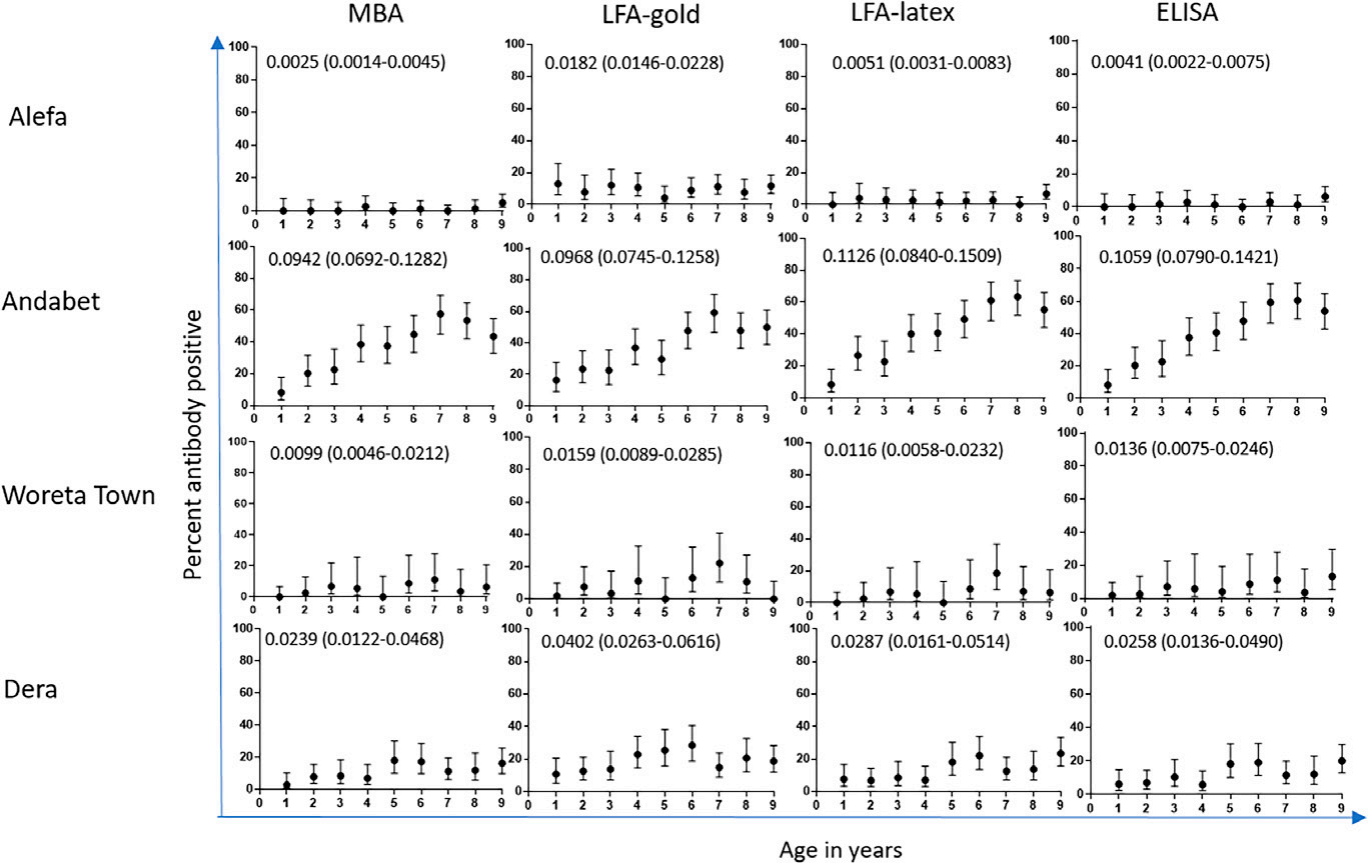
Percent antibody positive by age. Seroconversion rates (SCR) and 95% CI in parenthesis on each graph. LFA = lateral flow assay; MBA = multiplex bead assay.

Figure 2 shows the percent antibody positive by year of age and the SCR estimates for each assay and district. The districts that had a TF prevalence > 5% (Andabet and Dera) had SCR > 0.02 by all assays. The districts that had TF prevalence < 5% (Alefa and Woreta Town) had an SCR < 0.015 by all assays except for LFA-gold.

### Performance of LFA and ELISA compared with MBA.

The kappa between readers for samples run by LFA-latex and LFA-gold is shown for each district in Table 3. The percent agreement between MBA and LFA-gold was 89.1% (95% CI 87.7–90.3), between MBA and LFA-latex was 96.8% (95% CI 96.0–97.5), and between MBA and ELISA was 97.4% (95% CI 96.7–98.0). The kappa between MBA and LFA-gold was 0.608 (0.564–0.653), between MBA and LFA-latex was 0.878 (0.850–0.906), and between MBA and ELISA was 0.899 (0.873–0.926). Samples with discordant results between the MBA and other assays fell closer to the MBA cutoff value for ELISA and LFA-latex (median MFI-bg of 729 and 516, respectively) than LFA-gold (median MFI-bg of 7).

**Table 3:** Inter-rater agreement as quantified by kappa for LFA-gold and LFA-latex by district. 95% CI in parenthesis

EU	LFA-gold (95% CI)	LFA-latex (95% CI)
Alefa	0.731 (0.599–0.820)	1.00 (1.00–1.00)
Andabet	ND	0.986 (0.972–1.00)
Woreta Town	ND	0.964 (0.892–1.00)
Dera	ND	0.960 (0.927–0.992)

EU = evaluation unit; LFA = lateral flow assay; ND = not done.

## DISCUSSION

Population-based serological surveys can give an indication of exposure to a pathogen that can be modeled to estimate transmission in that population. We have been investigating whether and how antibody testing can provide information about transmission of ocular Ct in children to help trachoma programs monitor endemic or previously endemic populations.

Each test platform has advantages. MBA has good reproducibility (Gwyn, submitted for publication) and can be multiplexed with other antigens to optimize integrated serosurveillance.^16^ Although this is not technically a gold standard test for antibodies against Pgp3, the strong performance of this test^17^ (Gwyn, submitted for publication) makes it a suitable comparator for other tests. The ELISAs against Pgp3 perform generally well compared with MBA, and the double antigen ELISA we use here shows good population-level agreement with MBA. Both of these tests also provide semi-quantitative data that may be useful for some analyses of antibody levels^18^ that the LFA does not provide. But the generally low cost, lack of instrument requirements, and ease of training and use make the LFA an appealing option for postvalidation surveillance where funding may be scarce or nonexistent.

The current study shows optimization of a rapid lateral flow-based test (Pgp3-LFA) in response to population-level serological data that contrasted greatly with other tests for this sample set. These data—particularly the LFA-gold panel in Figure 2—highlight the subjectivity of tests using chromogenic readouts rated by a person. For this study, the technician documented if they considered bands to be faint, very faint, or very very faint on a worksheet during the study. Although these notations were outside of protocol, they proved helpful in understanding the issues at hand, as these samples were routinely negative by other tests and most likely represented overcalling of positive tests. We posited that this occurred because running DBS on the LFA can lead to a pink smear. As a result, the red test line that appears with a colloidal gold developing reagent often needs to be differentiated from a light pink background. The test line for strongly positive specimens are easy to differentiate from the background, but for weakly positive specimens this can become challenging. For low-prevalence populations, the tendency to overcall tests as positive may be higher than others, because technicians may feel the need to look for positive specimens. In support of this, the differences in LFA-gold results compared with other tests in this study was most striking in the low-prevalence settings of Woreta Town and Alefa.

Although changing the LFA to a black test readout line greatly improved readability as seen by the excellent inter-rater agreement, there is still a great deal of subjectivity in using an LFA. Other steps that could be taken to improve test performance are to have multiple raters confirm the test classification, to use an automated reader, or to use an external control as a comparator. The primary advantages of a rapid LFA is speed and low cost. Because of this, using multiple raters in a population-based survey for every sample may not be an appealing option, but encouraging confirmatory readings for tests with borderline positives may be helpful. Use of automated readers has the potential to be high throughput but adds cost and would need to be customized for the test format. Lateral flow assays run with a defined concentration of an external control antibody could provide a visual threshold for test interpretation; a humanized monoclonal antibody against Pgp3 is currently under development. Because a rapid test such as this would require less training and instrumentation than ELISA or MBA, it may be desired by countries conducting surveillance. Investigating ways to standardize the classification of a test line will be critical for LFA use.

Integrating seroprevalence data by year of age has been used as a measure of cumulative exposure,^19^^–^^21^ which could prove especially useful for surveillance for a disease such as trachoma. Long-term pathology of trachoma requires multiple infections with Ct, perhaps > 100 over a lifetime.^22^ Catalytic models estimate the rate of seroconversion by using the relative prevalence of seropositive to seronegative individuals in a population over a period of time; in this case, using year of age as a proxy for time.^23^ Because these models use year of age as the denominator, seroprevalence in 1- and 2-year-olds contributes more heavily to the estimated SCR compared with older children. The data in Figure 2 show how this can lead to SCR values that contradict the visual “shape” of the age seroprevalence curve. For the LFA-gold data from Alefa, the age seroprevalence curve is generally flat (contrast this with the curves from Andabet) but is elevated on the *y* axis compared with the other tests for that district. The resulting SCR derived from the LFA-gold test in Alefa is therefore much higher than SCR derived from the other tests. Similar inconsistencies are seen with the LFA-gold data from Dera.

One limitation of this study and serological studies more generally is that the lack an external control makes evaluating antibody tests challenging (a notable exception are tests for tetanus and other vaccine-preventable diseases for which international standards exist). Blood from infection-positive individuals is not the most appropriate control for sensitivity for a test to measure antibodies, which circulate potentially months or years after an infection clears. For serology to be programmatically useful for trachoma, and more generally as an indicator for ongoing transmission, it will be critical to have standardized ways to evaluate test performance and metrics of transmission.

In the absence of such an external control or defined panel to validate the serology for trachoma surveillance, we have used epidemiological correlates to evaluate the performance of tests for Pgp3-specific antibodies. The current study adds to the existing data by establishing limitations of our LFA-gold test, describing an improved version of the LFA using a black detection line, and confirming the strong performance of the ELISA. Both seroprevalence and SCR estimated using ELISAs corresponded closely with MBA-derived estimates, and ELISAs are widely used in laboratories worldwide. Although the costs for ELISA testing would likely be higher than for LFA, this test could provide an option for serological testing in endemic countries should the limitations imposed by the LFA in terms of subjectivity and instability of SCR estimates prove difficult to overcome.

## References

[1] World Health Organization , 2020. WHO alliance for the global elimination of trachoma by 2020: progress report, 2019. Wkly Epidemiol Rec 95: 349–360.

[2] Martin DL , 2020. The use of serology for trachoma surveillance: current status and priorities for future investigation. PLoS Negl Trop Dis 14: e0008316. 32970672 10.1371/journal.pntd.0008316 PMC7514076

[3] Martin DL , 2015. Serology for trachoma surveillance after cessation of mass drug administration. PLoS Negl Trop Dis 9: e0003555. 25714363 10.1371/journal.pntd.0003555 PMC4340913

[4] Senyonjo LG , 2018. Serological and PCR-based markers of ocular *Chlamydia trachomatis* transmission in northern Ghana after elimination of trachoma as a public health problem. PLoS Negl Trop Dis 12: e0007027. 30550537 10.1371/journal.pntd.0007027 PMC6310292

[5] West SK Zambrano AI Sharma S Mishra SK Munoz BE Dize L Crowley K Gaydos CA Rotondo LA, 2017. Surveillance surveys for reemergent trachoma in formerly endemic districts in Nepal from 2 to 10 years after mass drug administration cessation. JAMA Ophthalmol 135: 1141–1146. 28973295 10.1001/jamaophthalmol.2017.3062 PMC5710394

[6] Martin DL , 2015. Serological measures of trachoma transmission intensity. Sci Rep 5: 18532. 26687891 10.1038/srep18532 PMC4685243

[7] Pinsent A , 2018. The utility of serology for elimination surveillance of trachoma. Nat Commun 9: 5444. 30575720 10.1038/s41467-018-07852-0 PMC6303365

[8] Goodhew EB Priest JW Moss DM Zhong G Munoz B Mkocha H Martin DL West SK Gaydos C Lammie PJ, 2012. CT694 and Pgp3 as serological tools for monitoring trachoma programs. PLoS Negl Trop Dis 6: e1873. 23133684 10.1371/journal.pntd.0001873 PMC3486877

[9] Gwyn S Cooley G Goodhew B Kohlhoff S Banniettis N Wiegand R Martin DL, 2017. Comparison of platforms for testing antibody responses against the *Chlamydia trachomatis* antigen Pgp3. Am J Trop Med Hyg 97: 1662–1668. 29016320 10.4269/ajtmh.17-0292 PMC5805053

[10] Gwyn S Mitchell A Dean D Mkocha H Handali S Martin DL, 2016. Lateral flow-based antibody testing for *Chlamydia trachomatis.* J Immunol Methods 435: 27–31. 27208400 10.1016/j.jim.2016.05.008

[11] Horner PJ Wills GS Righarts A Vieira S Kounali D Samuel D Winston A Muir D Dickson NP McClure MO, 2016. *Chlamydia trachomatis* Pgp3 antibody persists and correlates with self-reported infection and behavioural risks in a blinded cohort study. PLoS One 11: e0151497. 26974653 10.1371/journal.pone.0151497 PMC4790965

[12] Sun MJ Zambrano AI Dize L Munoz B Gwyn S Mishra S Martin DL Sharma S West SK, 2017. Evaluation of a field test for antibodies against *Chlamydia trachomatis* during trachoma surveillance in Nepal. Diagn Microbiol Infect Dis 88: 3–6. 28214223 10.1016/j.diagmicrobio.2017.01.004 PMC11025397

[13] Gwyn S Mkocha H Randall JM Kasubi M Martin DL, 2019. Optimization of a rapid test for antibodies to the *Chlamydia trachomatis* antigen Pgp3. Diagn Microbiol Infect Dis 93: 293–298. 30709562 10.1016/j.diagmicrobio.2018.11.001

[14] Gwyn S , 2021. Comparison of platforms for testing antibodies to *Chlamydia trachomatis* antigens in the Democratic Republic of the Congo and Togo. Sci Rep 11: 7225. 33790370 10.1038/s41598-021-86639-8 PMC8012353

[15] Nash SD , 2021. Population-based prevalence of *Chlamydia trachomatis* infection and antibodies in four districts with varying levels of trachoma endemicity in Amhara, Ethiopia. Am J Trop Med Hyg 104: 207–215. 33200728 10.4269/ajtmh.20-0777 PMC7790060

[16] Arnold BF Scobie HM Priest JW Lammie PJ, 2018. Integrated serologic surveillance of population immunity and disease transmission. Emerg Infect Dis 24: 1188–1194. 29912680 10.3201/eid2407.171928 PMC6038749

[17] Wiegand RE Cooley G Goodhew B Banniettis N Kohlhoff S Gwyn S Martin DL, 2018. Latent class modeling to compare testing platforms for detection of antibodies against the *Chlamydia trachomatis* antigen Pgp3. Sci Rep 8: 4232. 29523810 10.1038/s41598-018-22708-9 PMC5844876

[18] Arnold BF van der Laan MJ Hubbard AE Steel C Kubofcik J Hamlin KL Moss DM Nutman TB Priest JW Lammie PJ, 2017. Measuring changes in transmission of neglected tropical diseases, malaria, and enteric pathogens from quantitative antibody levels. PLoS Negl Trop Dis 11: e0005616. 28542223 10.1371/journal.pntd.0005616 PMC5453600

[19] Corran P Coleman P Riley E Drakeley C, 2007. Serology: a robust indicator of malaria transmission intensity? Trends Parasitol 23: 575–582. 17988945 10.1016/j.pt.2007.08.023

[20] Drakeley CJ , 2005. Estimating medium- and long-term trends in malaria transmission by using serological markers of malaria exposure. Proc Natl Acad Sci USA 102: 5108–5113. 15792998 10.1073/pnas.0408725102 PMC555970

[21] Golden A , 2016. Analysis of age-dependent trends in Ov16 IgG4 seroprevalence to onchocerciasis. Parasit Vectors 9: 338. 27296630 10.1186/s13071-016-1623-1 PMC4907250

[22] Gambhir M , 2009. The development of an age-structured model for trachoma transmission dynamics, pathogenesis and control. PLoS Negl Trop Dis 3: e462. 19529762 10.1371/journal.pntd.0000462 PMC2691478

[23] Stewart L , 2009. Rapid assessment of malaria transmission using age-specific sero-conversion rates. PLoS One 4: e6083. 19562032 10.1371/journal.pone.0006083 PMC2698122

